# Contextualized analysis of a needs assessment using the Theoretical Domains Framework: a case example in endocrinology

**DOI:** 10.1186/1472-6963-14-319

**Published:** 2014-07-24

**Authors:** Patrice Lazure, Robert C Bartel, Beverly MK Biller, Mark E Molitch, Stephen M Rosenthal, Judith L Ross, Brock D Bernsten, Sean M Hayes

**Affiliations:** 1 AXDEV Group Inc., 210–8, Place du Commerce, Brossard, QC J4W 3H2, Canada; 2 The Endocrine Society, 8401 Connecticut Avenue, Suite 900, Chevy Chase, MD, USA; 3 Neuroendocrine Unit, Harvard Medical School/Massachusetts General Hospital, 55 Fruit St, Bulfinch 457B, Boston, MA 02114-2096, USA; 4 Division of Endocrinology, Metabolism and Molecular Medicine, Northwestern University, Feinberg School of Medicine, 645 N. Michigan Ave., Suite 530, Chicago, IL 60611-3008, USA; 5 Department of Pediatrics, University of California, 513 Parnassus Ave Room S-672, Box 0434, San Francisco, CA 94143-0434, USA; 6 Pediatric Endocrinology, Jefferson Medical College, 1025 Walnut St Ste 726, Philadelphia, PA 19107-6799, USA; 7 Town and Country Pediatric Medical Associates, 3838 California St., #111, San Francisco, CA 94118, USA

**Keywords:** Theoretical domains framework, Application of educational research, Needs assessment, Mixed–methods, Behavioral change techniques, Knowledge translation, Endocrinology, Adult growth hormone deficiency, Acromegaly, Pediatric growth disorders

## Abstract

**Background:**

The Theoretical Domains Framework (TDF) is a set of 14 domains of behavior change that provide a framework for the critical issues and factors influencing optimal knowledge translation. Considering that a previous study has identified optimal knowledge translation techniques for each TDF domain, it was hypothesized that the TDF could be used to contextualize and interpret findings from a behavioral and educational needs assessment. To illustrate this hypothesis, findings and recommendations drawn from a 2012 national behavioral and educational needs assessment conducted with healthcare providers who treat and manage Growth and Growth Hormone Disorders, will be discussed using the TDF.

**Methods:**

This needs assessment utilized a mixed-methods research approach that included a combination of: [a] data sources (Endocrinologists (n:120), Pediatric Endocrinologists (n:53), Pediatricians (n:52)), [b] data collection methods (focus groups, interviews, online survey), [c] analysis methodologies (qualitative - analyzed through thematic analysis, quantitative - analyzed using frequencies, cross-tabulations, and gap analysis). Triangulation was used to generate trustworthy findings on the clinical practice gaps of endocrinologists, pediatric endocrinologists, and general pediatricians in their provision of care to adult patients with adult growth hormone deficiency or acromegaly, or children/teenagers with pediatric growth disorders. The identified gaps were then broken into key underlying determinants, categorized according to the TDF domains, and linked to optimal behavioral change techniques.

**Results:**

The needs assessment identified 13 gaps, each with one or more underlying determinant(s). Overall, these determinants were mapped to 9 of the 14 TDF domains. The Beliefs about Consequences domain was identified as a contributing determinant to 7 of the 13 challenges. Five of the gaps could be related to the Skills domain, while three were linked to the Knowledge domain.

**Conclusions:**

The TDF categorization of the needs assessment findings allowed recommendation of appropriate behavior change techniques for each underlying determinant, and facilitated communication and understanding of the identified issues to a broader audience. This approach provides a means for health education researchers to categorize gaps and challenges identified through educational needs assessments, and facilitates the application of these findings by educators and knowledge translators, by linking the gaps to recommended behavioral change techniques.

## Background

Knowledge translation (KT) theories recognize that healthcare environments are complex and dynamic systems, with multiple factors and stakeholders of influence [1,2]. Over the years, various models and theories of behavioral, psychological, and/or socio-cultural investigation have been used in KT research. This article proposes using one such model, the Theoretical Domains Framework (TDF), to contextualize and interpret findings from a behavioral and educational needs assessment, a study aiming to identify the clinical behavior gaps (defined as the difference between "what is" – what the healthcare providers are doing - and "what should be" – the best practices) [3] and educational needs of a given healthcare provider population. The following introduction will briefly present the TDF, before providing details of the clinical background of the needs assessment, which was conducted with healthcare providers who treat and manage patients with growth and growth hormone (GH) disorders.

### The theoretical domains framework

To provide structure to the emergence of multiple KT models, Michie and colleagues [4] detailed the results of a consensus process that aimed to unify multiple health, social and psychological theories relevant to KT research. Three groups of contributors, comprised of specialists in psychological theory, health services research, and health psychology were involved in a 6-phase process. The result was the Theoretical Domains Framework (TDF), a set of 12 domains of behavior change that was subsequently updated to 14 [5]. These 14 domains provide a comprehensive framework for critical issues and factors influencing optimal KT and educational research incorporating individual, interpersonal, systems and contextual variables, that is not tied to any specific medical approach or therapeutic area.

To date, the TDF has been deployed in two ways [6]. The first use is referred to as Theoretical Domains Interviewing (TDI) [7,8], and is applied to interviews with groups needing performance change. Group members report barriers and enablers that are analyzed using TDF domains, enabling optimal design of an intervention to implement the needed performance change. Secondly, studies have identified optimal behavioral change techniques for each domain, in order to support educators in the selection of the most appropriate technique for KT, performance change and/or educational interventions. Of note, Michie and colleagues [9] used a consensus approach, where four experts in health and clinical psychology identified and classified 118 behavioral change techniques into 35 categories, and provided a consensus-based recommendation for each possible combination of a TDF domain and a behavioral change technique. The four possible recommendations were: 1) should be used; 2) should not be used; 3) disagreed (for combinations where consensus could not be reached); or 4) uncertain (when not enough evidence was available in the literature to take a position).

Recently, French and colleagues [10] integrated the two aforementioned uses to propose a systematic four-step method for the development of behavior-focused interventions based on the TDF: 1) identify the behavior to be changed; 2) identify barriers and enablers; 3) identify appropriate behavior change techniques; and 4) assess changes in behavior.

The authors of this paper hypothesized that the TDF could be used as part of an analysis plan to contextualize and interpret findings from a needs assessment. More specifically, we postulated that the TDF would provide a rigorous structure by which results of a content-specific needs assessment could be explained to a wider audience, drive effective evidence-based KT, behavioral change and educational strategies, and facilitate links between the needs assessment and the first three steps of French’s development method [9]. This approach would allow the findings and recommendations of a TDF-mapped needs assessment to be readily interpreted by health educators without detailed content knowledge. To illustrate this hypothesis, the authors will present and discuss a TDF post-hoc analysis of research findings drawn from a 2012 national performance and educational needs assessment conducted with healthcare providers who treat and manage patients with growth and growth hormone (GH) disorders.

### Clinical background of the needs assessment

The Endocrine Society is a professional association of over 16,000 physicians and scientists involved in the study and treatment of endocrine disorders. As part of its mission, it strives to keep its members current on the management and treatment of endocrine disorders, including growth and GH disorders, a complex set of conditions that can impact children, adolescents, and adults. Adult growth hormone deficiency (AGHD) typically causes increased fat mass, elevated LDL cholesterol levels, reduced bone mineral density, higher fracture rates, decreased muscle mass, energy and quality of life [11]. Adults with excessive secretion of GH have acromegaly, a chronic disease that can cause high blood pressure, type 2 diabetes mellitus, increased risk of cardiovascular disease, and arthritis [12]. Pediatric growth disorders (PGD) may have multiple etiologies, including familial short stature, constitutional delay, chronic non-endocrine disease, endocrine disorders, nutritional deficits, and a variety of miscellaneous causes including some genetic syndromes [13]. Based on the etiology of the growth disorder, GH may be considered an appropriate therapy. However, there are many issues for endocrinologists to consider in regards to initiation and continuation of therapy: effectiveness and safety of medical therapy, patient satisfaction, cost-benefit analysis of continuing therapy, and transition from pediatric to adult care [11,14,15].

Thus, The Endocrine Society undertook a behavioral and educational needs assessment to better understand the clinical challenges faced by physician providers in this field (pediatric and adult endocrinologists, general pediatricians), to determine their knowledge, skill, competency and performance gaps, and to identify system issues that interfere with effective diagnosis, management and treatment of patients with growth and GH disorders. The purpose of this needs assessment was to provide evidence-based recommendations on how to design and deploy effective educational interventions for physician providers involved in the care of patients with GH or growth disorders (pediatric and adult endocrinologists, general pediatricians). The objective of this paper is to determine if contextualizing the findings of this needs assessment according to the TDF, in order to provide a broader and more generalizable meaning to them, will simplify identification of optimal educational interventions for these endocrine care providers. We present the methodology of the behavorial and educational needs assessment, analysis of findings, contextualization and categorization according to the TDF, and recommendations for optimal educational strategies using the TDF categorization.

## Methods

**Figure 1:**
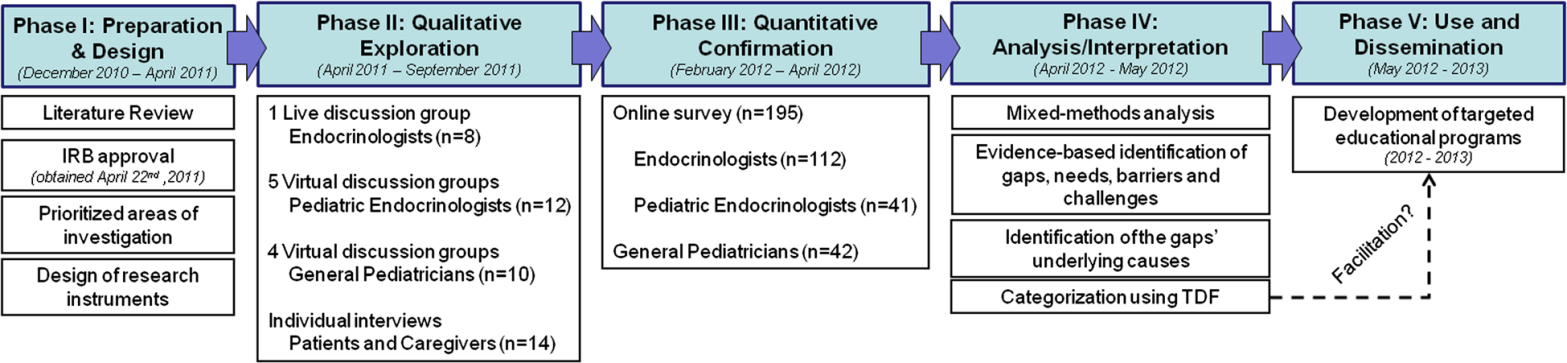
Overview of methodology.

Given the complexity of evaluating, diagnosing, and managing patients with growth and GH disorders, a mixed-methods (qualitative and quantitative) behavioral and educational needs assessment was selected. This approach incorporates the collection - and integrated analysis - of qualitative and quantitative data, drawing upon the strengths and depth of qualitative exploratory data collection and the analytic power of quantitative data collection [16,17]. To improve trustworthiness of findings [17,18], triangulation was employed, a technique which consists of combining several research methodologies to respond to the same research question [19]. For this study, triangulation of approaches (qualitative, quantitative), data collection methods (focus groups, survey, cases), and data sources (i.e., pediatric endocrinologists, general pediatricians, adult endocrinologists) were incorporated. In addition, a small sample of patients and caregivers was also interviewed, providing complementary insights and perspectives on the challenges experienced by physicians, and thus indirectly, on physicians’ needs. An overview of the research methodology, including implementation timelines, is provided in Figure 1. Clinical aspects of the research design were overseen by a steering committee of 5 Faculty, including 2 endocrinologists, 2 pediatric endocrinologists, and 1 general pediatrician (co-authors BMKB, MEM, SMR, JLR and BDB). All steering committee members are nationally-recognized experts in growth and GH disorders and hold leadership positions within their institutions, participate on national treatment guideline committees, and are part of Editorial Boards for peer-reviewed publications.

### Sampling and inclusion criteria

All participants were required to be actively practicing in the United States. Pediatric endocrinologists were required to be providing care to children and/or teenagers with PGD, representing a caseload of at least 10% of their practice. General pediatricians were required to be practicing in primary care settings and to be providing care to children and/or teenagers with PGD, but no minimal percentage of caseload was required. Adult endocrinologists were required to be providing care to a minimum of 5 patients with AGHD or acromegaly per year. In addition, specific criteria (region, clinical setting, years of experience) were identified and employed in purposive sampling. More precisely, a combination of criterion sampling (where individuals meeting a specific criteria are recruited) [20], and maximum variation sampling (where individuals covering the spectrum of perspectives are recruited) [20], was used, with the aim of obtaining a broad spectrum of perspectives that did not significantly deviate from the characteristics of the national healthcare provider population [17,20].

For patients and caregivers, inclusion criteria comprised a diagnosis of AGHD, acromegaly or PGD within a minimum of six months prior to participation, and the requirement to be actively followed by either an endocrinologist or a pediatric endocrinologist.

### Recruitment and ethical considerations

Recruitment methods included faxes and e-mails to potential participants from The Endocrine Society national membership database (endocrinologists and pediatric endocrinologists), the Magic Foundation membership (patients and caregivers), an ad published in the Hormone Hotline Newsletter, and a list purchased from an external supplier (general pediatricians). Independent ethical approval was obtained from Institutional Review Board Services (IRB Services, Aurora, Ontario, Canada) to ensure informed consent, protection and confidentiality of participants, and respect of national and international guidelines and policies on human subject research [21,22]. Informed consent was obtained online from all participants. All participants received ethically acceptable levels of compensation (i.e., market fair, but not enough to create coercion) for their time, based on the extent of their participation (survey or focus group).

### Qualitative data collection

Findings from a literature review informed the development of guides used in semi-structured focus groups (2 hours; pediatric and adult endocrinologists) and semi-structured phone interviews (45 minutes; general pediatricians). The two different collection methods were selected to maximize the contribution of each group of participants. Focus groups were selected for the groups of physicians, allowing for dynamic and interactive conversation and debate around controversies, while individual interviews allowed for the facilitators to respect each patient/caregiver’s level of comfort with the topic [20,23]. Three different guides were used for the physicians’ discussions in order to fit each provider’s professional role. These guides included open-ended questions and probes to stimulate discussion around challenges and barriers to the application of best practices in the selection of a treatment plan for patients with GH deficiency or excess. A sample of the qualitative questions is presented in Table 1. Each focus group and interview was audio-recorded with the consent of the participant(s), and was carried out by experienced facilitators (co-authors PL and SMH). The findings obtained from a preliminary analysis of the qualitative data (see analysis plan below for more details) were used to lead the quantitative survey development, and select the precise behaviors of interest to be investigated through the quantitative phase (see Table 1) [24].

**Table 1:** Examples of questions used in the qualitative exploratory investigation, and examples of themes emerging from qualitative data

**Broad domains of qualitative exploration**	**Example of exploratory qualitative questions (no precise behaviors targeted)**	**Example of themes emerging from qualitative data (i.e., behaviors to be further investigated)**
Diagnosis	What challenges do you experience in screening and diagnosing growth disorders?	Screening and management of GH deficiency
		Diagnosis of GH excess
Treatment	What type of challenges (if any) do you experience in your treatment choices?	Treatment criteria and guideline application in PGD
		Multi-modal therapy for GH excess
		Clinical decision-making regarding the choice of treatment in acromegaly
Management	What (if any) are your challenges in effectively managing or monitoring your patients with growth disorders?	Ensuring smooth transition of care from childhood to adulthood
		Referrals from primary care pediatricians to Pediatric Endocrinologists

### Quantitative data collection

Questions were designed to either validate qualitative findings through targeted questions, or to further investigate the behaviors of interest determined by the exploratory qualitative phase (see Table 1). The resulting survey was developed as a series of quantitative questions using multiple Likert-type scales, addressing participants’: a) current level of knowledge, skill, and confidence (1 = low; 5 = high); b) desired level of knowledge, skill, and confidence with regards to their role as an endocrinologist, pediatric endocrinologist or general pediatrician (1 = low; 5 = high); c) perception of barriers to optimal care (1 = not a barrier; 5 = a major barrier); and d) clinical behaviors and attitudes (1 = almost never; 5 = almost always). These scales have been used in previous needs assessments in other therapeutic areas, and have shown to provide usable data on the gaps of the targeted providers [25-27]. Table 2 presents sample questions used in the survey. Clinical cases selected by the faculty steering committee were embedded in the survey to assess clinical decision-making leading to treatment choices. The use of case-based questions has been demonstrated to be a valid tool for investigating potential clinical practice gaps [28].

**Table 2:** Examples of questions and items used in the quantitative phase of the study

**Category**	**Examples of questions**	**Scale anchors**	**Examples of items**
a) current level of knowledge, skill, and confidence	Please select the number that best describes how you currently evaluate your level of [*knowledge/confidence/skill*] concerning each issue.	1 = low; 5 = high	The usefulness of using priming substances in GH stimulation test
			Considering all co-morbidities in the selection of a treatment plan
b) desired level of knowledge, skill, and confidence with regards to their role	Please indicate what you think is the required level of [*knowledge/confidence/skill*] with regards to your role in the care for patients with [*GH deficiency/GH excess/PGD*]	1 = low; 5 = high	
			Negotiating patients’ resistance to daily injection treatment
c) perception of barriers to optimal care	Please indicate to what extent you think each of the following is a barrier for you in providing optimal care to patients *[GH deficiency/GH excess/PGD]*.	1 = not a barrier; 5 = a major barrier	Worries about possible long-term side-effects of growth hormone therapy
			Patients’ resistance to daily injections
d) clinical behaviors and attitudes	Please indicate to what extent you are following each clinical practice behavior in providing care to patients with [*GH deficiency/GH excess/PGD*]	1 = almost never; 5 = almost always	I generally avoid using growth hormone stimulation tests because I have no confidence in their value
			When I propose a treatment to a patient, I discuss the potential impact on his/her quality of life

The surveys included questions common to all three specialties and specific questions for each specialty, ensuring adaptation to each provider’s role. Fourteen case studies were also included in the survey, four of which were focused on GH deficiency, three on acromegaly and seven on pediatric growth disorders: two were exclusively designed for pediatric endocrinologists, two were designed for general pediatricians, and three were designed for both. Each case was design to investigate how providers would manage a specific and realistic clinical situation that faculty hypothesized to be particularly challenging.

Clinical cases were reviewed by all members of the steering committee to ensure their validity, including their agreement on which answer choices constituted the best practice answer. Surveys were pilot tested internally with an informed audience for face validity and duration, and adjustments were made to ensure clarity and respect of the allotted time.

### Analysis plan

Facilitators completed a standardized form after each focus group and interview to evaluate data quality. Transcription of selected focus groups and interviews was performed, based on these evaluations, until saturation was achieved. Experienced qualitative researchers (including co-authors PL and SMH) conducted the analysis using N-Vivo 7.0 software^a^. The approach included three steps: data familiarization (where the researcher immerses him/herself in the data), data coding (where the researcher codes and classifies data according to broad areas of interest), and theme identification (where the researcher identifies specific themes with substantial data emerging from the domains of exploration investigated) [29,30]. Theme identification was validated among researchers and discrepancies were resolved through discussions until concordance was achieved. Concordance was achieved in all cases. The themes identified were then used to determine the behaviors of interest to be investigated through the quantitative survey (Table 1).

Quantitative data from the first two series of survey questions (current and desired levels of knowledge, skill, and confidence) were analyzed using a method called gap analysis, where the current levels are subtracted from the desired levels to obtain a measure of the gap between "what is" and "what should be" [3]. Using SPSS 12.0 software^b^, frequencies and cross-tabulations were obtained for the two remaining series of questions (perception of barriers, and clinical behaviors and attitudes). Respondents’ answers to each of the clinical cases were compared with optimal answers, as identified by treatment guidelines and faculty experts.

The aforementioned approaches, methods and sources of data were triangulated to generate trustworthy and reliable findings on the challenges experienced by endocrinologists, pediatric endocrinologists, and general pediatricians in their provision of care to adult patients with AGHD or acromegaly, or children/teenagers with PGD. The different data sources were analyzed for complementarities, similarities and contradictions. The interdisciplinary group of researchers collectively reviewed each finding, and agreed on a final list of substantial gaps.

### Post-hoc categorization of needs assessment findings using the TDF

Categorization of the identified gaps was then undertaken using the TDF. Each gap was categorized in one or more of the 14 TDF domains, by an expert researcher. The revised version of the TDF [4] was preferred to its original version [3] because the authors of the revision demonstrated sufficient evidence-based support for the adjustments they proposed. A two-step process was performed by 1) breaking down each gap into its key underlying determinants, as identified in the needs assessment, and 2) matching each of the gap’s determinants with the most appropriate domain(s) of the TDF. For example, a gap on proactive patient-provider communication was broken down into 5 key underlying determinants, and each determinant was matched with a specific TDF domain: Lack of knowledge of existing tools to facilitate communication ("Domain 1 – Knowledge"); Lack of skills in communicating efficiently with patients ("2 – Skills"); Lack of confidence in discussing specific topics with patients ("4 - Beliefs about Capabilities"); Belief that patient will inquire if he/she has questions or concerns ("5 – Optimism"); and Underestimation of impacts of miscommunications ("6 - Beliefs about Consequences"). This analysis process was validated amongst researchers, and discrepancies were resolved through discussions until concordance was achieved.

### Linking of needs assessment findings to behavior change techniques, through the TDF

Once educational gaps impacting the delivery of care related to PGD and adult GH disorders were broken down into key underlying determinants and classified into TDF domains, they were subsequently linked to consensus-based behavior change techniques, using the four possible consensus-based recommendation levels proposed by Michie et al. [8]: 1) should be used; 2) should not be used; 3) disagreed, or 4) uncertain. The use of the TDF-based map allowed identified challenges to be linked to behavior change techniques that educational specialists could use to bridge the educational gaps.

## Results

### Sample size and demographics

Responses from 225 providers were assessed; 30 from the qualitative assessment and 195 from the quantitative assessment. While the response rate could not be obtained for the qualitative phase due to multiple channels of participant recruitment, it was estimated to be at 12.9% for the quantitative phase, with a completion rate of 3.8%. Table 3 provides the sample distribution and demographic characteristics of the participants. Solo or group practice settings accounted for 73% of pediatric endocrinologists and general pediatricians, and 64% had over 10 years of clinical experience. Providers practiced predominantly in urban (45%) or suburban (49%) settings. In addition, 15 patients or caregivers participated in the qualitative phase of the study.

**Table 3:** Sample distribution and characteristics for both study phases for each category of healthcare provider, as well as for patients and caregivers

**Healthcare Providers**	**Phase 1: Qualitative (n = 30)**	**Phase 2: Quantitative (n = 195)**	**TOTAL (n = 225)**
**Specialty**			
Endocrinologists	8	112	120 (53.3%)
Pediatric Endocrinologists	12	41	53 (23.6%)
General Pediatricians	10	42	52 (23.1%)
**Gender**			
Men	20	108	128 (57.1%)
Women	10	86	96 (42.9%)
**Region**			
Northeast	13	53	66 (29.3%)
Midwest	6	43	49 (21.8%)
South	5	59	64 (28.4%)
West	6	40	46 (20.4%)
**Practice setting**			
Solo or group practice	20	144	164 (72.9%)
Community hospital or clinic	6	27	33 (14.7%)
Government hospital	0	11	11 (4.9%)
Academic Medical Center	4	5	9 (4.0%)
Other	0	8	8 (3.6%)
**Years of practice**			
0-5 years of practice	1	35	36 (16.0%)
6-10 years	3	41	44 (19.6%)
11-20 years	13	71	84 (37.3%)
21-30 years	10	38	48 (21.3%)
More than 30 years	3	10	13 (5.7%)
**Practice location**			
Urban	10	92	102 (45.3%)
Suburban	18	93	111 (49.3%)
Rural	2	10	12 (5.3%)
**Patients & Caregivers**			
Adult patients with GH deficiency	5	N/A	5 (35.7%)
Caregivers of child with growth disorder	9		9 (64.3%)
**Gender**			
Male	1	N/A	1 (7.7%)
Female	13		13 (92.3%)
**Age of child**			
Age of child = 0–6 years old	1	N/A	1 (11.1%)
Age of child = 7–12 years old	4		4 (44.4%)
Age of child = 13–18 years old	4		4 (44.4%)
**Region**			
Northeast	1	N/A	1 (7.7%)
Midwest	1		1 (7.7%)
South	6		6 (42.9%)
West	6		6 (42.9%)

### Identified gaps, challenges and barriers in the needs assessment

A summary of the thirteen substantive gaps, challenges and barriers that were identified is presented in Table 4. A selection of illustrative quotes are available in Additional file 1. Although the study included other topics, only results that led to the identification of educational needs are presented. Because the study aimed to inform future educational initiatives, it focused on elements of care needing improvement at the detriment of areas where care was excellent.

**Table 4:** List of substantive gaps, challenges and barriers identified (items in bold are the focus of this article), with their corresponding TDF domain(s)

**#**	**Challenge**	**AGHD**	**Acromegaly**	**PGD**	TDF Domains*
1	Challenges communicating with patients, especially in overcoming patients’ barriers and resistances	X	X	X	1, 2, 4, 5, 6
2	Challenges with the treatment decision-tree	X	X	—	1, 2, 4, 9, 10
3	Challenges associated with the transition from childhood to teen years, to adulthood	—	—	X	1, 2, 3, 4, 6, 11
4	Insurance companies processes interfere with clinical decisions	X	X	X	11
5	Use of appropriate materials to support patient education	X	X	X	1, 11
6	Referrals between general pediatricians and pediatric endocrinologists (timeliness, appropriate pre-testing)	—	—	X	1, 4, 6
7	Perceptions of GH therapy	X	—	—	6
8	Lack of clarity in roles and responsibilities	X	X	X	3
9	Application of diagnostic tests	X	—	X	2, 4
10	Lack of screening by primary care	—	X	—	6
11	Identifying tests needed for at risk co-morbidities	—	—	X	2, 6
12	Presenting Treatment as optional	X	—	—	6
13	Inconsistencies between labs (system)	X	X	X	11

* "*1 – Knowledge*"; "*2 – Skills*"; "*3 - Social/Professional Role and Identity*"; "*4 - Beliefs about Capabilities*"; "*5 – Optimism*"; "*6 - Beliefs about Consequences*"; "*7 – Reinforcement*"; "*8 – Intentions*"; "*9 – Goals*"; "*10 - Memory, Attention, and Decision Processes*"; "*11 - Environmental Context and Resources*"; "*12 - Social Influences*"; "*13 – Emotion*"; and "*14 - Behavioral Regulation*".

### Categorization of educational and performance gaps by TDF

Each of the thirteen gaps, challenges and barriers were linked post-hoc to one or more TDF domain, based on the details of their underlying determinants. Results of TDF-based categorization of the challenges identified by the needs assessment are presented in the right-hand column of Table 4. Potential determinants were revealed from 9 of the 14 TDF domains. The domain "*6 - Beliefs about Consequences*" was identified as a contributing determinant to 7 of the 13 challenges. Five of the gaps could be related to the "*2 - Skills*" domain, while three were linked to the "*1 -Knowledge*" domain.

In order to respect space limitations, only the three most substantive challenges will be detailed and discussed in this manuscript, specifically those related to (A) Communication, (B) the complexities of the AGHD and acromegaly treatment decision-trees*,* and (C) Transitions from childhood to adolescence and adulthood. Details of TDF-based categorization of these three selected challenges can be found in Table 5 and are discussed in detail below. The three challenges presented cover all 9 TDF domains where determinants were identified, and should be sufficient to assess the value of TDF as a categorization framework for educational and behavioral needs assessment. For the ten remaining challenges identified, details of the TDF-based categorization are available in Additional file 2.

**Table 5:** List of the three most substantive gaps, challenges and barriers identified, with results of their secondary analysis using TDF domains

**#**	**Challenge**	**TDF Domain**	**Underlying determinant of the Challenge**
1	Challenges in overcoming patient barriers and resistance	"1 – Knowledge"	Lack of knowledge of tools to facilitate patient – provider communication
		"2 – Skills"	Lack of communication skills
		"4 - Beliefs about Capabilities"	Lack of confidence
		"5 – Optimism"	Underestimation of impacts of miscommunications
		"6 - Beliefs about Consequences"	
2	Challenges with the treatment decision-tree	"1 – Knowledge"	Extent to which short-term adverse side-effects compare to long term benefits of treatment in patients with AGHD
		"2 – Skills"	Challenge in balancing long term benefits of treatment with short-term adverse effects
		"4 - Beliefs about Capabilities"	Confidence issues
		"9 – Goals"	Prioritization between proximal goal of avoiding side effects and distal goal of avoiding long-term consequences of disorder
		"10 - Memory, Attention, and Decision Processes"	Specific challenges in the clinical reasoning process
3	Challenges associated with the transition from childhood to teen years, to adulthood	"1 – Knowledge"	Consequences of inappropriate cessation of GH therapy
		"2 – Skills"	Lack of skills in addressing compliance during teenage years
		"3 - Social/Professional Role and Identity"	Lack of clarity on which tests should be done at primary care levels and which ones should be done by specialists
		"4 - Beliefs about Capabilities"	General pediatricians preferring to refer because of lack of confidence
		"6 - Beliefs about Consequences"	Underestimation of impact of growth hormone treatment cessation
		"11 - Environmental Context and Resources"	Lack of adult-treating endocrinologists forcing pediatric endocrinologists to keep patients under their management longer

#### Challenges in overcoming patient barriers and resistance

In AGHD, endocrinologists reported challenges in effectively overcoming patient barriers and resistance related to treatment, with 4 underlying determinants being mapped to 5 of the TDF domains (Table 6). Specifically, endocrinologists reported that management of their patients’ resistance to daily injection treatment (43%), and their communication skills in addressing patient resistance (41%) are important barriers to care. In addition, 26% of endocrinologists reported not always recommending daily GH injections to avoid possible patient resistance, even if it is perceived as the best treatment option. In acromegaly, 47% of endocrinologists also see a barrier in the patient’s resistance to injection treatment with a medication that treats the excessive GH secretion. Ten percent of endocrinologists have recommended another treatment plan to avoid possible patient resistance, even if the injections are the best treatment for that particular patient. Endocrinologists reported gaps in their skills to communicate the value of treatment in AGHD (31%), to explain the long-term benefits versus the short-term adverse side-effects to asymptomatic patients (44%), and to explain AGHD to patients in lay language (37%). Forty-eight percent of endocrinologists stated they have seen patients abandon treatment, due to their challenges in effectively communicating the benefits of GH therapy in the absence of visible change. A gap in knowledge of existing tools to facilitate patient-provider communication with AGHD patients was reported by 58% of endocrinologists.

**Table 6:** **Recommendations on behavior change techniques (as per Michie 2008**[4]** see original article for description of each technique category) for each underlying determinant within the three most substantive gaps, challenges and barriers identified**

**TDF Domain**	**Identified challenges (underlying determinant)**	**Techniques Recommendations *(All other techniques are in the "agreed non-use" category)***
1 - Knowledge	Challenges in overcoming patient barriers and resistance (Lack of knowledge of tools to facilitate patient – provider communication)	Agreed use: Information regarding behavior, outcome
		Uncertain: goal/target specified: behavior or outcome//Persuasive communication
	Challenges with the treatment decision-tree (Extent to which short-term adverse side-effects compare to long term benefits of treatment in patients with AGHD)	Disagreement: Personalized message//Homework
	Challenges associated with the transition from childhood to teen years, to adulthood (Consequences of inappropriate cessation of GH therapy)	
2 -Skills	Challenges in overcoming patient barriers and resistance (Lack of communication skills)	Agreed use: goal/target specified: behavior or outcome//Monitoring//Self-monitoring//rewards & incentives//Graded tasks//problem-solving, decision-making, goal-setting//Rehearsal//Modeling, demonstration of behavior//Homework//perform behavior in different settings//problem-solving
	Challenges with the treatment decision-tree (balancing long term benefits of treatment with short-term adverse effects)	
	Challenges associated with the transition from childhood to teen years, to adulthood (Lack of skills in addressing compliance during teenage years)	Uncertain: Stress management//Planning, implementation
		Disagreement: Coping skills//Role-play//Feedback//Shaping of behavior
3 - Social/Professional Role and Identity	Challenges associated with the transition from childhood to teen years, to adulthood (Lack of clarity on which tests should be done at primary care levels and which ones should be done by specialists)	Agreed use: Social process of encouragement, pressure, support
		Uncertain: Contract//rewards & incentives//problem-solving, decision-making, goal-setting//Role-play//Environmental changes//Persuasive communication//Personalized message//feedback
		Disagreement: Information regarding behavior, outcome//Modeling, demonstration of behavior//Cognitive restructuring
4 - Beliefs about Capabilities	Challenges in overcoming patients’ barriers and resistances (Lack of confidence)	Agreed use: Self-monitoring//Graded tasks//problem-solving, decision-making, goal-setting//Social process of encouragement, pressure, support//Feedback//self-talk//Motivational interviewing
	Challenges with the treatment decision-tree (Lack of confidence)	
	Challenges associated with the transition from childhood to teen years, to adulthood (General pediatricians preferring to refer because of lack of confidence)	Uncertain: Monitoring//rewards & incentives//Coping skills//Rehearsal//Stress management//Information regarding behavior, outcome//Personalized message//Experiential//Use of imagery//perform behavior in different settings//Shaping of behavior//Relapse prevention//Identify-prepare for difficult situations, problems
		Disagreement: -
5 - Optimism	Challenges in overcoming patients’ barriers and resistances (Underestimation of impacts of miscommunications)	Agreed use: Self-monitoring//Graded tasks//problem-solving, decision-making, goal-setting//Social process of encouragement, pressure, support//Feedback//self-talk//Motivational interviewing
		Uncertain: Monitoring//rewards & incentives//Coping skills//Rehearsal//Stress management//Information regarding behavior, outcome//Personalized message//Experiential//Use of imagery//perform behavior in different settings//Shaping of behavior//Relapse prevention//Identify-prepare for difficult situations, problems
		Disagreement: Role-Play//Persuasive communication//Modeling, demonstration of behavior//Homework//Personal experiments//Cognitive restructuring
6 - Beliefs about Consequences	Challenges in overcoming patients’ barriers and resistances (Underestimation of impacts of miscommunications)	Agreed use: Self-monitoring//Persuasive communication//Information regarding behavior, outcome//Feedback
	Challenges associated with the transition from childhood to teen years, to adulthood (Underestimation of impact of growth hormone treatment cessation)	Uncertain: Monitoring//rewards & incentives//Role-play//Personalized message//Personal experiments
		Disagreement: goal/target specified: behavior or outcome//Homework//Motivational interviewing//Cognitive restructuring
9 - Goals	Challenges with the treatment decision-tree (prioritization between proximal goal of avoiding side effects and distal goal of avoiding long-term consequences of disorder)	Agreed use: Social process of encouragement, pressure, support//Modeling, demonstration of behavior
		Uncertain: Monitoring//rewards & incentives//Role-play//persuasive communication//homework
		Disagreement: Contract
10 - Memory, Attention, and Decision Processes	Challenges with the treatment decision-tree (Specific challenges in the clinical reasoning process)	Agreed use: Self-monitoring//Planning, implementation//Prompts, triggers, cues
		Uncertain: Monitoring//rewards & incentives//Graded tasks//problem-solving, decision-making, goal-setting//stress management//Rehearsal//Personalized message//Personal experiments//feedback//self-talk//use of imagery//perform behavior in different settings//Time management
		Disagreement: Environmental changes
11 - Environmental Context and Resources	Challenges associated with the transition from childhood to teen years, to adulthood (Lack of adult-treating Endocrinologists forcing Pediatric Endocrinologists to keep patients under their management longer)	Agreed use: Environmental changes
		Uncertain: Prompts, triggers, cues
		Disagreement: -

Pediatric endocrinologists and general pediatricians identified a need for improved skills and confidence in communicating with parents. Specifically, providers expressed difficulty in dealing with parents seeking treatment for their child who was short but considered within a normal height range. Conversely, providers also struggle with parents who did not perceive their child’s growth deficiency as problematic, and were in denial. Additional specific barriers that providers find difficult to overcome include parents’ lack of understanding regarding their child’s disorder, and parents’ misinformation, through non-medical sources, about treatment outcomes and long-term side effects of GH treatment.

The challenges in overcoming patient barriers and resistance were linked to five TDF domains, through four underlying determinants. The first determinant reported was communication skills, which was linked to TDF domain "*2 - Skills*". Many of the detailed results within that challenge were linked to the provider’s professional confidence, a construct that is part of TDF domain "*4 - Beliefs about Capabilities*". A tendency was observed for providers to downplay the consequences of some of their communication challenges, not always realizing that a lack of communication can lead to a misinformed patient who may then make a misinformed choice. This tendency is linked to both "*6 - Beliefs about Consequences*", and "*5 - Optimism*". Finally, there is an aspect of this issue that can be linked to "*1 - Knowledge*", specifically regarding patient – provider communication tools.

#### Challenges with the AGHD and Acromegaly treatment decision-tree

Five underlying determinants of endocrinologists’ gap in AGHD treatment decisions were identified and linked post-hoc to 5 TDF domains (Table 5). In AGHD, endocrinologists reported lack of confidence in treating AGHD once diagnosed (39%), determining if adverse side-effects outweigh long term benefits of treatment (55%), and adjusting dosage of therapy to minimize adverse side-effects (51%).

Similarly in acromegaly, confidence gaps were reported in making the appropriate clinical decisions when managing uncured post-operative acromegaly (61%), deciding how long to wait before trying another medical option that provides biochemical control (64%), and recommending alternate treatment options, should the first medical treatment selected not provide biochemical control (67%).

In addition, endocrinologists reported gaps in their skills to select the appropriate treatment option when managing uncured post-operative acromegaly (49%), and in recommending alternate treatment options, should the first medical treatment selected not provide biochemical control (53%).

Providers also expressed challenges in monitoring benefits and side-effects associated with GH therapy in AGHD (46%), and in assessing the impact of GH replacement on quality of life (52%). Additionally, 48% of endocrinologists reported a gap in their knowledge of the extent to which short-term side-effects compare to long term benefits of treatment in patients with AGHD.

The underlying determinants of the AGHD and acromegaly treatment decision-trees challenges were linked to five of the TDF domains. Several specific issues were linked to the provider’s "*2 – Skills*", and several others were linked to professional confidence, a construct that is part of TDF domain "4 *- Beliefs about Capabilities*". The issues described above will affect decision-making, a construct that the TDF authors have classified into the "*10 - Memory, Attention, and Decision Processes*" domain. Finally, one issue of "*1 – Knowledge*" was reported by the endocrinologists (comparison of short-term side-effects to long term benefits of treatment in patients with AGHD). In-depth analysis can also link this same issue to the "*9 – Goals*" domain, through the two constructs of distal vs. proximal goals and goal priority.

#### Challenges associated with the transition from childhood to teen years to adulthood

Six underlying determinants were identified for challenges associated with the transition from childhood to teen years to adulthood, and were linked post-hoc to 6 of the TDF domains (Table 5). Of note, pediatric endocrinologists reported a need to improve their ability to respond to caregivers’ concerns about the impact of PGD on puberty. Two thirds of providers perceived the facilitation of treatment compliance following the onset of puberty as a frequent struggle. Adult endocrinologists expressed similar issues in taking on young adult patients, highlighting their need for enhanced competencies in addressing management of GHD with that sub-population. Additionally, providers identified that negotiating conflicts between teenagers and their parents with regards to treatment choice, and supporting patients in coping with the possibility of lifelong treatment, also required improved competencies. Furthermore, half of pediatric endocrinologists reported continuing to care for patients with GHD into their mid-20s, either because they perceived it was better for the patient, or because they could not find an adult endocrinologist that would take on the patient. A related treatment gap in endocrinologists’ knowledge of the consequences of cessation of GH therapy in patients who remain deficient was also identified. Only 58% of endocrinologists selected the correct answer for the case developed on this issue highlighting an important knowledge gap.

The underlying determinants of the challenges associated with the transition periods were linked to six TDF domains. There is an issue of "*1 – Knowledge*" reported by the endocrinologists (consequences of cessation of GH therapy in patients who remain deficient), that also linked to the domain of "*6 - Beliefs about Consequences*". Facilitation of treatment compliance and several other issues can be linked to domain "*2 – Skills*". There are also underlying determinants that can be attributed to "*3 - Social/Professional Role and Identity*", as there is a lack of clarity on which tests should be done at primary care levels and which ones should be done by specialists. General pediatricians preferring to refer due to lack of confidence can be linked to TDF domain "*4 - Beliefs about Capabilities*". Finally, "*11 - Environmental Context and Resources*" is also a determinant, specifically when pediatric endocrinologists report managing patients for longer because they were unable to find an adult endocrinologist who would treat their patient for GHD.

## Discussion

The authors hypothesized that Theoretical Domains Framework (TDF) could provide a valuable structure for the post-hoc categorization and interpretation of findings from an educational needs assessment conducted in growth and GH disorders, as a means to provide deeper understanding of the identified gaps, and readily utilizable direction to plan educational activities. This approach can provide insights to educators to develop effective and targeted KT and educational interventions, and to more effectively interpret complex content-specific findings without requiring expertise in that content area.

A total of 13 challenges impacting the delivery of care related to PGD and adult GH disorders were broken down into key underlying determinants, classified into TDF domains, and subsequently linked to consensus-based behavior change techniques, using Michie et al.’s [4] proposed TDF-based map. Table 6 presents the recommendations from TDF-based map for the three most substantive challenges identified in this study, broken down into their underlying determinants.

The process of categorizing the behavioral and educational gaps according to TDF was clear, and no gaps were left without a linked domain. Two pairs of domains appear slightly more difficult to distinguish: "*5 – Optimism*" and "*6 - Beliefs about Consequences*", as well as "*8 – Intentions*" and "*9 – Goals*". However, the definitions provided by Cane and colleagues [2] helped resolve and classify the gaps appropriately. Five domains were not used in this classification process: "*7 – Reinforcement*"; "*8 – Intentions*"; "*12 - Social Influences*"; "*13 – Emotion*"; and "*14 - Behavioral Regulation*". That does not mean that there are no causal links between gaps and those domains, but rather that no evidence allowed the authors to make such links.

The value behind categorizing behavioral and educational gaps according to TDF lies in the identification of multiple variables that might have been otherwise neglected, had the specific gap not been analyzed and classified into underlying determinants. In addition, dissecting gaps into their underlying determinants for classification into the 14 TDF domains opens the door to a broad inventory of evidence-based behavior change techniques that could be used to address the specific needs identified. As such, it allows educators who are not experts in a specific domain (e.g., are not PGD/AGHD/Acromegaly specialists) to apply the findings. Linking each TDF domain to multiple behavior change techniques facilitates efficient identification of optimal learning techniques for a given gap by educators and KT strategists. Alternatively it allows combination of multiple techniques, optimizing the possibility of meeting the learning preferences of each learner. It is hypothesized that presenting needs assessment findings mapped to TDF domains could facilitate use by educators, as it traces a path between the first three steps of French’s four-step systematic method for development of behavioral change interventions [7]. In addition, the range of behavioral change techniques available through TDF categorization of gaps enables educators to develop learning curricula that follow educational logic, and can affect behaviors in an optimal sequence. For example, TDF categorization for the AGHD and acromegaly treatment decision-tree delineates that learning curricula should first address clinicians’ knowledge of short-term side effects and long term benefits of treatment through informational, didactic sessions. This should be followed by interventions such as problem-solving and graded tasks that target their skills in balancing those short and long term consequences of treatment (Table 6). This allows clinicians to concentrate on treatment goal prioritization through behavioral modeling exercises. Finally, and perhaps most importantly, while the TDF facilitates communication and understanding of issues influencing optimal KT in growth and GH disorders in the context of this manuscript, because it is not tied to any specific approach, it does not require any understanding or knowledge of endocrinology as a subject matter.

While most medical societies develop interventions to address the full scope of medical knowledge in their specialty, relatively few regularly produce interventions addressing the full range of Accreditation Council of Graduate Medical Education competencies [31] (e.g. interpersonal and communication skills, professionalism, practice-based learning and improvement, systems based practice). As medical specialty societies strive to enhance educational value and measure the efficacy of educational interventions on clinician performance and patient health outcomes, they must engage in more robust needs assessment methodologies that not only identify gaps, but contextualize their underlying determinants, and facilitate translation of the findings into KT or educational activities.

TDF-framed needs assessment outcomes lend integrity and urgency to investment in producing new educational interventions for clinicians treating growth and GH disorders. For example, these findings highlight the need to address the challenges inherent to the transition of care for patients with GHD from the pediatric to adult endocrinologist. While traditional didactic interventions raise awareness of the need for additional vigilance by providers, this report suggests the opportunity for resources, such as clinical practice guidelines specifically addressing transitions of care for all the providers along this continuum. In addition, the findings place new emphasis on addressing physician to patient (or caregiver) communication, and suggest the opportunity to develop talking guides for all providers, patients and their caregivers. Likewise, distinct educational interventions for adolescent patients and their caregivers are being considered.

### Limitations

Categorization using TDF was a post-hoc analysis to better understand findings from a behavioral and performance needs assessment that had not been designed with TDF in mind. Despite efforts on the researchers’ part to the contrary, classification of the different causal variables of the gaps into TDF domains remains partly subjective. In addition, participants of the behavioral and performance needs assessment may have provided subjective interpretations of their contextual issues. Triangulation was used to strengthen the trustworthiness of the findings and to limit the subjectivity inherent to self-assessment. There is a possibility of self-selection bias, as participation in this study was voluntary. However, selective sampling was used to increase probability of having a sample that is representative of the targeted population. Finally, the relatively small sample size of patients and caregivers is not an important limitation to the study, considering they were not a direct target population, but rather their involvement was to enrich the mixed-methods triangulated approach.

## Conclusions

Results from this national needs assessment illustrate the links that can be made between behavioral and educational needs assessment findings and TDF. The question remains as to whether TDF model should be used a priori (i.e. in the planning phases of assessments of gaps) or a posteriori (i.e. in the interpretation phases of needs assessments). While both approaches seem to be suitable, the selection of either approach would depend on the objectives and scope of the initiative. A knowledge translator or educator who is seeking to further his understanding of a specific gap may want to consider using the a priori approach through a TDF methodology for instance, while another who is interested in exploring challenges and barriers in a therapeutic area may want to conduct a posteriori TDF analyses following a broader needs assessment.

Educational specialists who are preparing needs assessments should be aware of new developments and theories in the fields KT and behavioral change. For the educational cycle to reach its optimum potential, there needs to be continuity between those who assess needs and those who address the needs (i.e. education and KT/behavioral change specialists).

### Endnotes

^a^QSR International, Cambridge, MA.

^b^SPSS, Chicago, IL.

## Abbreviations

AGHD: Adult growth hormone deficiency; GH: Growth hormone; GHD: Growth hormone disorders; KT: Knowledge translation; PGD: Pediatric growth disorder; TDF: Theoretical domains framework; TDI: Theoretical domains interviewing.

## Competing interests

The authors declare that they have no competing interest.

## Authors’ contributions

PL co-led the deployment of the study, designed the data collection instruments, carried out the initial analyses and led the interdisciplinary interpretation of findings. He drafted the main part of the initial manuscript. RB co-led the deployment of the study, critically reviewed data collection instruments and participated in the interdisciplinary interpretation of findings. BMKB acted as the chair of the Faculty Steering Committee. She contributed cases for the online survey, critically reviewed data collection instruments and participated in the interdisciplinary interpretation of findings. MEM, SMR, JLR and Dr. BDB acted as members of the Faculty Steering Committee. They oversaw clinical aspects of the research design, contributed cases for the online survey, critically reviewed data collection instruments and participated in the interdisciplinary interpretation of findings. SMH conceptualized, designed and supervised the deployment of the study, and presented the findings to the other members of the collaborative after each phase. All authors provided a critical review of the drafted manuscript and approved the final manuscript as submitted.

## Pre-publication history

The pre-publication history for this paper can be accessed here:

http://www.biomedcentral.com/1472-6963/14/319/prepub

## Supplementary Material

Additional file 1: Table S1 Selection of exemplary quotes. This file includes a table providing readers with exemplary quotes from the qualitative assessment, classified by the gaps, challenges and barriers identified. Click here for file

Additional file 2: Table S2 Detailed analysis of the gaps, challenges and barriers identified. This file includes a table listing the thirteen gaps, challenges and barriers identified, with results of their secondary analysis using TDF domains. Click here for file
