# Supplementary material for: Contextualized analysis of a needs assessment using the Theoretical Domains Framework: a case example in endocrinology
Source: BMC Health Serv Res. 2014 Jul 24;14:319. doi: 10.1186/1472-6963-14-319 (PMC4123497; doi:10.1186/1472-6963-14-319)
Supplement: Additional file 1: Table S1 — Selection of exemplary quotes. This file includes a table providing readers with exemplary quotes from the qualitative assessment, classified by the gaps, challenges and barriers identified. [file 1472-6963-14-319-S1.pdf]

Additional file 1:

Detailed analysis of the gaps, challenges and barriers identified

Table S1: List of the thirteen gaps, challenges and barriers identified, with results of their secondary analysis using TDF domains.

#	Challenge	TDF Domain	Underlying determinant of the Challenge
1	Challenges in overcoming patients' barriers and resistances	See main text of manuscript.	
2	Challenges with the treatment decision-tree	See main text of manuscript.	
3	Challenges associated with the transition from childhood to teen years, to adulthood	See main text of manuscript.	
4	Insurance companies processes interfere with clinical decisions	"11 - Environmental Context and Resources"	Insurance companies not following clinical guidelines in setting the requirements to approve medications
5	Use of appropriate materials to support patient education	"1 – Knowledge"	Lack of knowledge of existing patient education tools to provide patients and caregivers
		"11 - Environmental Context and Resources"	Lack of available appropriate materials to support patient education.
6	Referrals between general pediatricians and pediatric endocrinologists (timeliness, appropriate pre-testing)	"1 – Knowledge"	Knowledge of optimal timing of referral
			Knowledge in the screening and testing procedures for growth hormone deficiency in children
		"4 - Beliefs about Capabilities"	Confidence in the screening and testing procedures
		"6 - Beliefs about Consequences"	Lack of awareness on consequences of under- / over- referral
7	Perceptions of GH therapy	"6 - Beliefs about Consequences"	Concerns over long-time side-effects of growth hormone therapy
8	Lack of clarity in roles and responsibilities	"3 - Social / Professional Role and Identity"	Lack of clarity in the professional boundaries between the roles of each provider
9	Application of diagnostic tests	"2 – Skills"	Challenges with selecting and interpreting growth hormone stimulation test
		Lack	Lack of confidence in selecting the appropriate form of growth hormone stimulation test
			Lack of confidence in formulating a differential diagnosis between the different pediatric growth disorders
10	Lack of screening by primary care	"6 - Beliefs about Consequences"	Lack of awareness on consequences of under-screening
11	Identifying tests needed for at risk co-morbidities	"2 – Skills"	Challenges with selecting the proper test to assess at-risk co-morbidities
		"6 - Beliefs about Consequences"	Lack of awareness on consequences of not appropriately testing for at-risk co-morbidities

#	Challenge	TDF Domain	Underlying determinant of the Challenge
12	Presenting Treatment as optional	“6 - Beliefs about Consequences”	Perception that GH treatment is optional since AGHD is generally not life-threatening
13	Inconsistencies between labs	“11 - Environmental Context and Resources”	Lack of consistency between results obtained by different laboratories (i.e., low inter-lab reliability)
