# Supplementary material for: Contextualized analysis of a needs assessment using the Theoretical Domains Framework: a case example in endocrinology
Source: BMC Health Serv Res. 2014 Jul 24;14:319. doi: 10.1186/1472-6963-14-319 (PMC4123497; doi:10.1186/1472-6963-14-319)
Supplement: Additional file 2: Table S2 — Detailed analysis of the gaps, challenges and barriers identified. This file includes a table listing the thirteen gaps, challenges and barriers identified, with results of their secondary analysis using TDF domains. [file 1472-6963-14-319-S2.pdf]

Additional file 2:
Selection of exemplary quotes

Table S2: Exemplary quotes, classified by the gaps, challenges and barriers identified.

#	Challenge	Exemplary quote(s)
1	Challenges in overcoming patients' barriers and resistances	"*To ask them to take a shot every night because their bone densities are lower, their lipids are a little off; I have a hard time talking them into it.*"<br>- Endocrinologist<br><br>"*I have about 10, 10%, 10 maybe 15% of hypopituitary on growth hormone. Those are the ones who have some symptoms, who don't feel well. But the others have tried; some I have tried to convince, they don't, they don't want it. Then I stopped it.*"<br>- Endocrinologist
2	Challenges with the treatment decision-tree	"*I've had a few patients and developed diabetes on growth hormones. I'm pretty sure it was a growth hormone. Then you had to make the decision do you stop the growth hormone or just keep them on growth hormone and treat the diabetes. So there's a lot of complexity and synergies*".<br>- Endocrinologist<br><br>"*And the other issue is some of these people may not complain, but they may have … osteoporosis* […] *When you put the, you know a kind of case pathway, particularly to growth hormone deficiency which might improve it*".<br>"*But there are other pathways to deal with, with osteoporosis* […] *There are others, other treatments, it's much easier to handle. I mean, what we are, I mean the realities while we're faced with hurdles, we sometimes choose easier pathways. I mean just to be honest*".<br>- Interaction between 2 endocrinologists<br><br>"[…] *you're dealing with these medical therapies that are only partially effective. You're not totally sure what criterion to use to decide if the patient really is curative afterward. You know, if you get progression, you know, where do you go?* […] *I mean there are just many, just real legitimate clinical challenges to managing uncured post-opt acromegalic*".<br>- Endocrinologist
3	Challenges associated with the transition from childhood to teen years, to adulthood	*"But they've* [teens] *hated doing it* [GH injections] *anyway and, they've only done it because their parents have been riding them. And now they're in college and you know there's no one riding them so why should they do it".*<br>– Endocrinologist<br><br>*"They* [the pediatrician and the pediatric endocrinologist] *are not giving me any really specific answers, just really vague. I'm concerned at this point that my son has not went* [sic] *into puberty on his own; he's 13 years old and they have told me that that's a little late for him to not have begun puberty".*<br>- Caregiver of child with PGD
4	Insurance companies processes interfere with clinical decisions	"*That's the way it works. I mean the insurance companies probably sit in meetings like this and scheme up the ways to slow down. I'm serious about this, I believe that they do, I believe that these things are, are consciously in there, they're creating on purpose. […] And I, short of a clinical solution, I don't know that there's a solution to that*".<br>- Endocrinologist

#	Challenge	Exemplary quote(s)
		"*We have this sort of disconnect between poor growth as an issue and growth hormone tests as a mechanism to limit reimbursement. So that's very problematic when you start dealing with the guidelines because insurance companies just redefine whatever they want*".<br>- Pediatric Endocrinologist
5	Use of appropriate materials to support patient education	"*We didn't get any reading material or anything like that from the Doctors, from the Endocrinologist about growth disorder. I had to do all that research on my own."*<br>- Caregiver of child with PGD
6	Referrals between general pediatricians and pediatric endocrinologists (timeliness, appropriate pre-testing)	*"One of the things that stand out to me is my lack of knowledge, specifically about certain growth disorders. I am somewhat challenged by knowing if I am waiting too long, or when I do order a test, what does the result really mean".*<br>- Primary Care Pediatrician
9	Application of diagnostic tests	"*I felt my own practice evolve away from using stimulation tests, because I find the challenge with them is they are not reproducible, they are not reliable, and they could be potentially dangerous. So one of the challenges is that I tend not to do stim tests* […]".<br>- Pediatric Endocrinologist<br><br>"*I do feel that stim test has definite limitations […] our guidelines are not so well defined and our stim tests are not accurate*".<br>- Pediatric Endocrinologist
12	Presenting Treatment as optional	"*I think growth hormone is still perceived as an optional hormone in adults*".<br>- Endocrinologist
13	Inconsistencies between labs	"*Run the assay in a different lab and lo and behold they don't need* [medication] *anymore. You know, I don't know what that's about. But that is certainly a challenge in managing these patients that I found*".<br>- Endocrinologist
